# Supplementary material for: Single Dose of Attenuated Vaccinia Viruses Expressing H5 Hemagglutinin Affords Rapid and Long-Term Protection Against Lethal Infection with Highly Pathogenic Avian Influenza A H5N1 Virus in Mice and Monkeys
Source: Vaccines (Basel). 2025 Jan 15;13(1):74. doi: 10.3390/vaccines13010074 (PMC11769126; doi:10.3390/vaccines13010074)
Supplement: Supplementary file 1 [file vaccines-13-00074-s001.zip › vaccines-3317434-supplementary.pdf]

Supplementary data for

Single Dose of Attenuated Vaccinia Viruses Expressing H5 Hemagglutinin Affords Rapid and Long-Term Protection Against Lethal Infection with Highly Pathogenic Avian Influenza A H5N1 Virus in Mice and Monkeys

Fumihiko Yasui [1,*], Keisuke Munekata [1], Tomoko Fujiyuki [2], Takeshi Kuraishi [3], Kenzaburo Yamaji [1], Tomoko Honda [1], Sumiko Gomi [1], Misako Yoneda [2], Takahiro Sanada [1], Koji Ishii [4], Yoshihiro Sakoda [5,6], Hiroshi Kida [6,7], Shosaku Hattori [3], Chieko Kai [2] and Michinori Kohara [1,*]

[1] Department of Microbiology and Cell Biology, Tokyo Metropolitan Institute of Medical Science, 2-1-6, Kamikitazawa, Setagaya-ku, Tokyo 156-8506, Japan
[2] Infectious Disease Control Science, Institute of Industrial Science, The University of Tokyo, 4-6-1, Komaba, Meguro-ku, Tokyo 153-8505, Japan
[3] Animal Laboratory of Injurious Animals, The Institute of Medical Science, The University of Tokyo, 802, Tean Sude, Setouchi-cho, Oshima-gun, Kagoshima 894-1531, Japan
[4] Center for Quality Management Systems, National Institute of Infectious Diseases, 4-7-1, Gakuen, Musashi-murayama, Tokyo 208-0011, Japan
[5] Laboratory of Microbiology, Faculty of Veterinary Medicine, Hokkaido University, Kita 18 Nishi 9, Kita-ku, Sapporo 060-0818, Japan
[6] Institute for Vaccine Research and Development (HU-IVReD), Hokkaido University, Sapporo 001-0021, Japan
[7] International Institute for Zoonosis Control, Hokkaido University, Sapporo 001-0020, Japan
* Correspondence: yasui-fm@igakuken.or.jp (F.Y.); kohara-mc@igakuken.or.jp (M.K.)

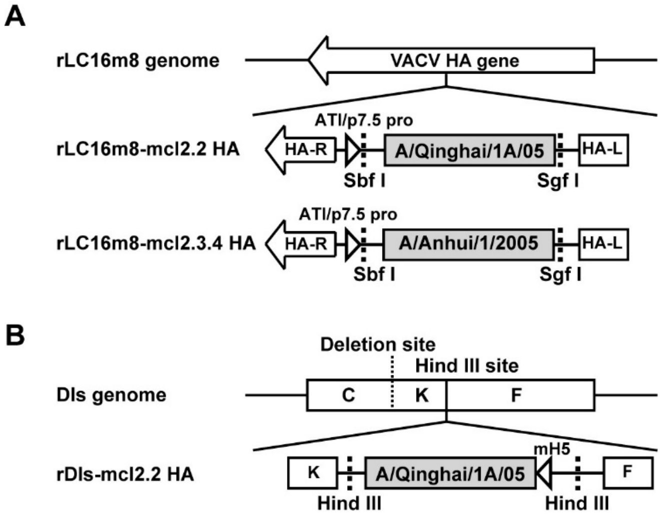

Figure. S1. Schematic diagram of two different types of recombinant VACV vector expressing avian influenza H5 HA protein. (A) rLC16m8-H5 HA viral vectors: complementary DNA (cDNA) fragment encoding either H5 clade 2.2 HA protein or H5 clade 2.3.4 HA protein was inserted into the HA gene of LC16m8 by homologous recombination using a transfer vector pBMSF7c. (B) rDIs-H5 HA viral vector: cDNA fragment encoding H5 clade 2.2 HA protein was inserted into the region Hind III site close to a large-scale deletion of DIs genome by homologous recombination using a transfer vector pUC/DIs.

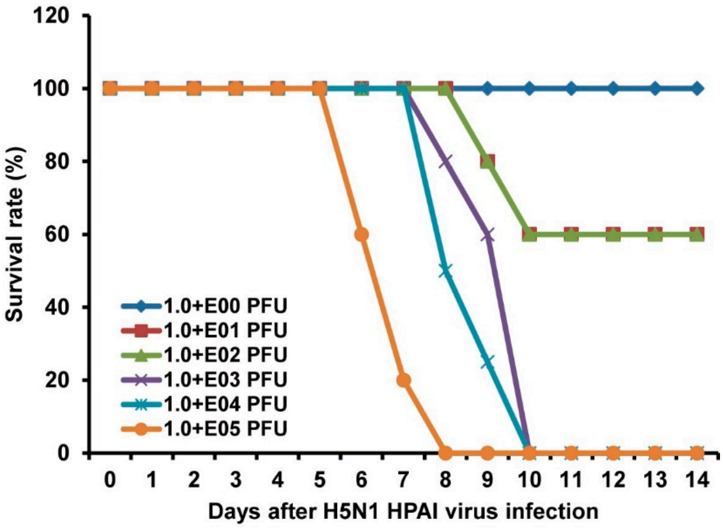

Figure. S2. Determination of the MLD_{50} of A/whooper swan/Hokkaido/1/2008 (H5N1) virus. Female BALB/c mice were inoculated intranasally with the indicated dose of A/whooper swan/Hokkaido/1/2008 (H5N1) virus and the survival rate was measured.

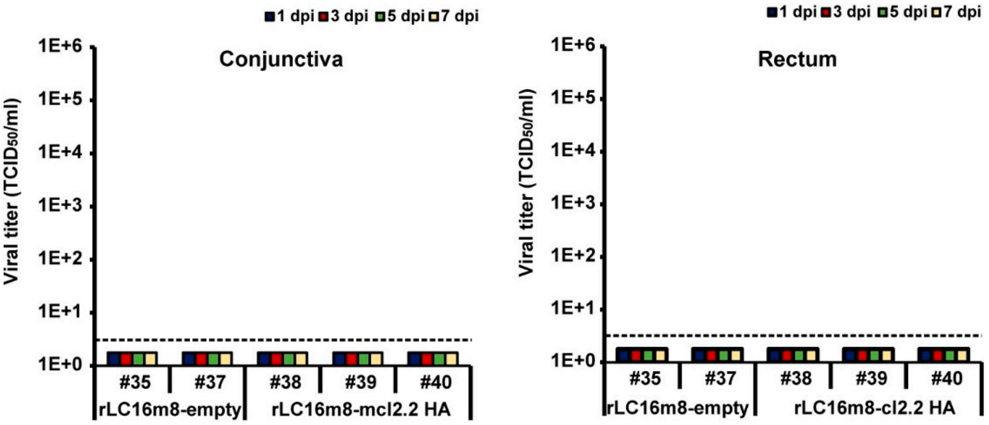

Figure. S2. Determination of virus titers in conjunctiva and rectal swab samples. Virus tier in conjunctiva (left panel) and rectal (right panel) swab samples were determined by $TCID_{50}$ assay using MDCK cells. The lower limit of detection (1.7 log units) is indicated by a horizontal dashed line.

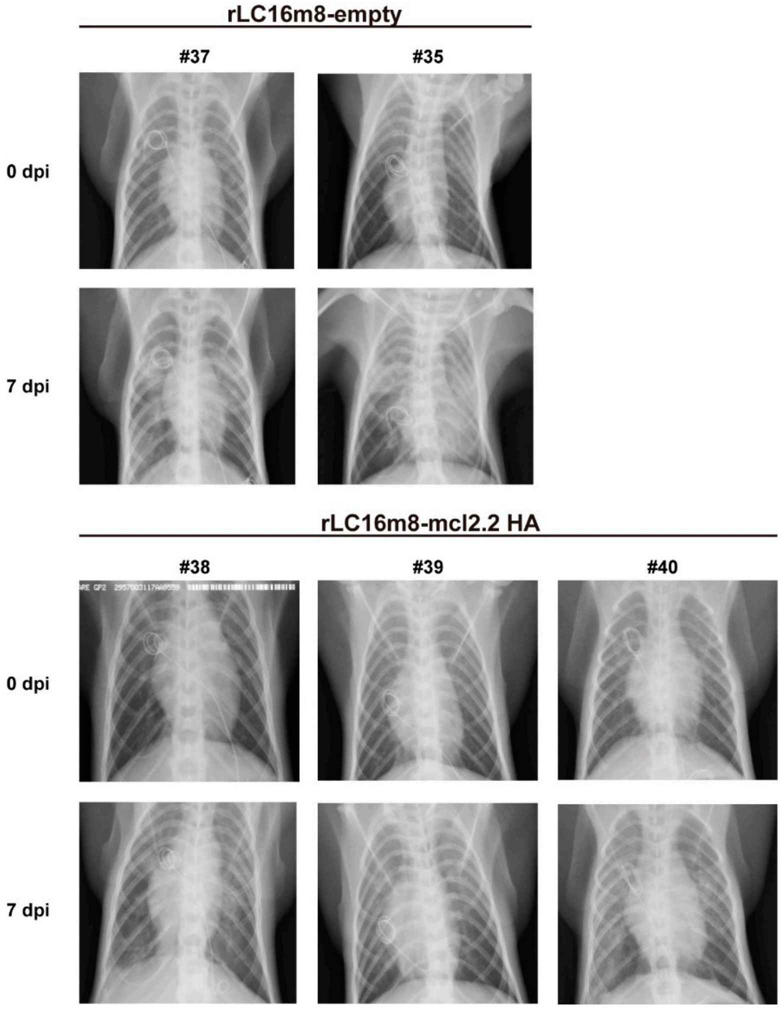

Figure. S4. Chest radiography of cynomolgus macaques infected with H5N1 HPAI virus. Twelve months post-vaccination, cynomolgus monkeys were infected with H5N1 HPAI virus A/whooper swan/Hokkaido/1/2008. Chest X-rays were taken at 0 and 7 dpi.

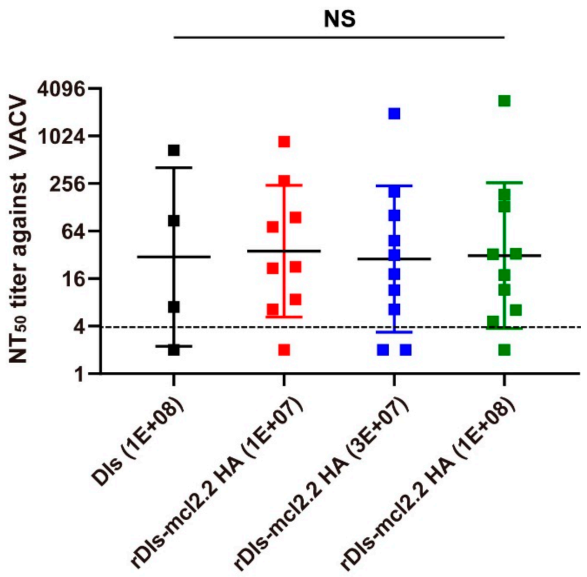

Figure. S5. Comparison of neutralizing antibody titer against VACV among 4 VACV-sensitized subgroups. The neutralizing antibody titer against VACV of all VACV-sensitized mice were determined. Mice were divided into 4 subgroups in which the neutralization titer against VACV was similar within each subgroup; 1 × 10^8 PFU of DIs, 1 × 10^7 PFU of rDIs-mcl2.2 HA, 3 × 10^7 PFU of rDIs-mcl2.2 HA, or 1 × 10^8 PFU of rDIs-mcl2.2 HA. *p* values were calculated via two-tailed non-paired One-way-ANOVA followed by Turkey's test. NS: not significant.
